# The Effect of Filamentous Turf Algal Removal on the Development of Gametes of the Coral *Orbicella annularis*

**DOI:** 10.1371/journal.pone.0117936

**Published:** 2015-02-06

**Authors:** Neidy P. Cetz-Navarro, Eugenio J. Carpizo-Ituarte, Julio Espinoza-Avalos, Guillermina Chee-Barragán

**Affiliations:** 1 El Colegio de la Frontera Sur, Chetumal, Quintana Roo, Mexico; 2 Posgrado en Oceanografía Costera, Instituto de Investigaciones Oceanológicas-Facultad de Ciencias Marinas, Universidad Autónoma de Baja California, Ensenada, Baja California, Mexico; 3 Instituto de Investigaciones Oceanológicas, Universidad Autónoma de Baja California, Ensenada, Baja California, Mexico; Leibniz Center for Tropical Marine Ecology, GERMANY

## Abstract

Macroalgae and filamentous turf algae (FTA) are abundant on degraded coral reefs, and the reproductive responses of corals may indicate sub-lethal stress under these conditions. The percentage of gametogenic stages (PGS) and the maximum diameter of eggs (MDE; or egg size) of *Orbicella annularis* were used to evaluate the effect of long- (7–10 months) and short-term (2.5 months) FTA removal (treatments T1 and T2, respectively) at both the beginning (May) and the end (August) of gametogenesis. Ramets (individual lobes of a colony) surrounded by FTA (T3) or crustose coralline algae (CCA; T4) were used as controls. The removal of FTA enhanced the development of gametes (i.e., a larger and higher percentage of mature gametes (PMG)) of *O. annularis* for T1 vs. T3 ramets in May and T1 and T2 vs. T3 ramets in August. Similar values of PGS and MDE between gametes from T3 and T4 in both May and August were unexpected because a previous study had shown that the same ramets of T4 (with higher tissue thickness, chlorophyll *a* cm^-2^ and zooxanthellae density and lower mitotic index values) were less stressed than ramets of T3. Evaluating coral stress through reproduction can reveal more sensitive responses than other biological parameters; within reproductive metrics, PGS can be a better stress indicator than egg size. The presence of turf algae strongly impacted the development of gametes and egg size (e.g., PMG in ramets with FTA removal increased almost twofold in comparison with ramets surrounded by FTA in August), most likely exerting negative chronic effects in the long run due to the ubiquity and permanence of turf algae in the Caribbean. These algae can be considered a stressor that affects coral sexual reproduction. Although the effects of turf algae on *O. annularis* are apparently less severe than those of other stressors, the future of this species is uncertain because of the combined impacts of these effects, the decline of *O. annularis* populations and the almost complete lack of recruitment.

## Introduction

Coral reefs are losing stony coral cover worldwide [1–6], and the space from which corals are lost is overgrown by macroalgae, hindering coral re-establishment and thus replacing the corals. Coral reefs in the Caribbean are among the most severely affected [7,8].

Assemblages of macroalgae and turf algae are associated with deleterious effects on corals, such as reduction of photosynthetic yields of symbiotic microalgae [9], inhibition of recruitment [10], reduction of the survivorship of juvenile corals [11], reduction in coral growth [12,13], and increased tissue mortality [12,14,15]. These algae have also become disease vectors [16]. In contrast, it is generally assumed that crustose coralline algae (CCA) have null or minimal detrimental effects on corals [17–20].

Corals are also subjected to a series of stressors that affect their reproduction. These stressors include bleaching, increases in water temperature, prolonged overcasts, decreases in seawater salinity, suspended sediments, injuries, and water eutrophication [21–27]. The response patterns of reproduction can be used as a sensitive indicator of sub-lethal stress [28,29] because coral reproduction appears to have a narrower tolerance to stress than other biological processes [23,30] such as maintenance (respiration, feeding, defense) and growth (including tissue regeneration).

The assemblages of macroalgae and turf algae also stress corals [31,32] and can affect their reproduction by reducing the diameter of eggs and the number of eggs formed in each mesentery (usually called gonad) [33], suppressing coral fecundity [34,35], and decreasing the amount of released larvae [36]. In contrast, the experimental removal of those algae can help eliminate coral stress [32] promoting higher numbers of eggs, larger eggs, an increased number of mesenteries with formed gametes (gonads) per polyp [33], and an increase in the number of larvae released [36]. However, little is known about the effect of algal turfs on the development of gametes of the most structurally important coral species on Caribbean reefs, including *Acropora palmata*, *Montastraea cavernosa*, and the *Orbicella annularis* species complex [37–39].

The common Caribbean coral *O*. *annularis* is a hermaphroditic species with an annual reproductive cycle that begins in May and ends in August-October with the release of gametes [34,40]. Gamete formation is asynchronous; oocyte formation begins in May, and spermary formation begins in June [34].

The aim of this study was to evaluate the effect of long- (7–10 months) and short-term (2.5 months) removal of filamentous turf algae (FTA) on the development of gametes of *O*. *annularis* at the beginning and the end of gametogenesis. Mats of FTA surrounding *O*. *annularis* included 96 taxa and were ~8 mm in height, with abundant sediment (grain size <0.3 mm) trapped within the mats [32]. The present study differs from [33] mainly because we used histological (vs. dissecting) procedures to obtain our data. Our approach allowed us to evaluate the development of gametes at different stages, and we included an additional algal assemblage (CCA) interacting with *O*. *annularis*.

## Materials and Methods

### Ethics Statement

The permit to collect *O*. *annularis* and FTA (DGOPA.10745.121009.3629) was furnished by the *Secretaría de Agricultura*, *Ganadería*, *Desarrollo Rural*, *Pesca y Alimentación* (SAGARPA, its acronym in Spanish).

### Study area

**Fig 1:**
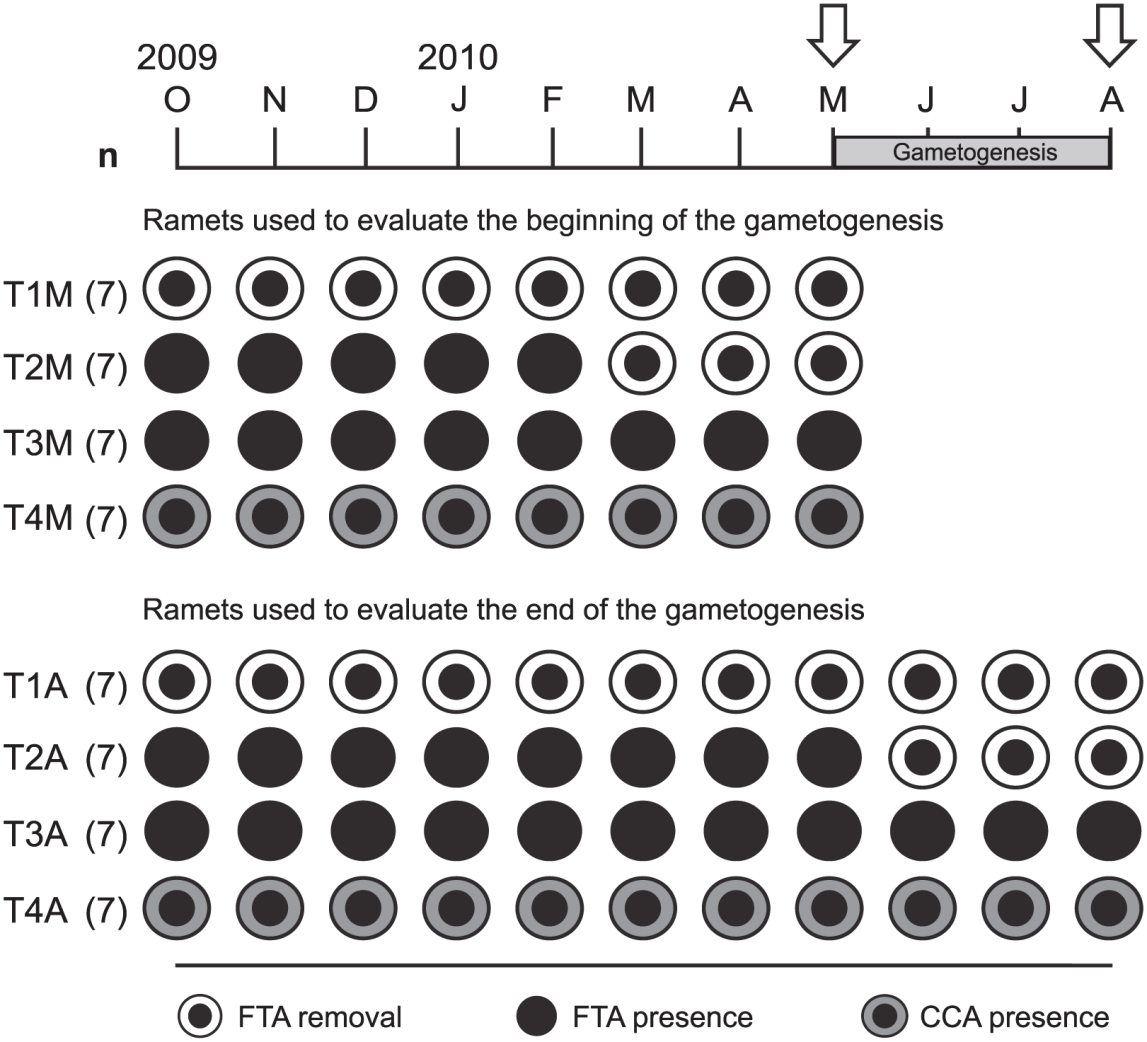
Experimental design used for *Orbicella annularis* ramets, with four treatments. The experiment included *O*. *annularis* ramets with filamentous turf algae removal from their periphery (FTA removal), ramets surrounded by FTA (FTA presence), and ramets with coralline crustose algae surrounding coral tissue (CCA presence). Experimental ramets were collected in May and August 2010 (arrows) to evaluate the development of gametes at the beginning (first four rows of treatments) and the end (last four rows of treatments) of *O*. *annularis* gametogenesis, respectively. Treatments: T1 = ramets with long-term FTA removal during 7 and 10 months before the beginning (T1M) and the end (T1A) of gametogenesis, respectively; T2 = ramets with short-term FTA removal during 2.5 months before the beginning (T2M) and the end (T2A) of gametogenesis; T3 = control ramets in permanent contact with FTA (T3M and T3A); and T4 = control ramets permanently surrounded by CCA (T4M and T4A).

The study site is part of the National Park *Arrecifes de Xcalak* (PNAX, its acronym in Spanish) and is located off the Xahuayxol coast, in the southern part of Quintana Roo, Mexico (18°30’ 11.9” N, 87°45’ 24.8” W); it is located in the reef lagoon, near the breaker zone, at ~1.5 m depth. Two types of settings for algal-coral interactions were observed in *Orbicella annularis* at the study site: ramets surrounded by FTA and ramets bordered by CCA (see Figs. 1A and 1B in [32]).

### Experimental design

Two experimental treatments (T) were established to evaluate the effects of long- and short-term removal of FTA (T1 and T2, respectively) in ramets of *O*. *annularis* at the beginning (May 25, 2010) and the end (August 24, 2010) of gametogenesis. Long-term FTA removal was conducted for 7 to 10 months (with monthly algal removal from October to December 2009 and algal removal every two weeks from January to August 2010), and short-term FTA removal was conducted for 2.5 months (with algal removal every two weeks). Ramets surrounded by FTA (T3) and surrounded by CCA (T4) were used as controls (Fig. 1). The FTA removal consisted of manually clearing (with the aid of a knife and wire brushes) an algal belt of ~3 cm width around the entire coral tissue periphery (> 40 cm) of the ramets (see Fig. 1C in [32]). The manual removal of FTA could have detrimental effects (damaging coral tissue). Accordingly, the clearance (see Fig. 1C in [32]), performed by NPCN and JEA, was made with great care, requiring many hours of diving. Additionally, we began by removing FTA from twice the number of ramets needed for sampling (see below). At the end, only those ramets showing the very least damage were selected for sampling. The effect of algal removal was not separated, but unmeasured effects did not interfere with the detection of differences among experimental treatments. In addition, the edge polyps of *O*. *annularis* are more active in growth and maintenance than in reproduction [34] and are less fecund [33] than those in the central part of the ramets. Most likely, this difference would decrease the effect of manipulation on the reproductive condition of fragments located ≥ 7 cm from the periphery of the ramet (see below). The removal of FTA surrounding ramets was performed in ramets within colonies of *O*. *annularis* with full reproductive capacity (ramets > 100 cm^2^ in colonies > 300 cm^2^) according to the description in [41]. A transparent flexible plastic circle of 100 cm^2^, placed at the top of the ramets, was used to select those whose area was greater than that of the circle.

The experimental design, which comprised four treatments, began on October 26, 2009 (month 0) and ended on August 24, 2010 (month 10) (Fig. 1): T1 consisted of long-term FTA removal and ended in May (T1M, 7 months of removal) and August (T1A, 10 months of removal) 2010. The T1 treatment was designed to evaluate the reproductive responses of the coral to the removal of FTA at the beginning and the end of *O*. *annularis* gametogenesis. T2 consisted of FTA removal on a short-term basis (2.5 months; T2), which was performed in two ramet groups, from March to May (T2M) and from June to August (T2A) 2010, to evaluate the reproductive condition at the beginning and the end of gametogenesis, respectively. The last two treatments, involving *O*. *annularis* ramets surrounded by FTA (T3) and by CCA (T4), were used as controls; both were collected in May (the beginning of gametogenesis; T3M and T4M, respectively) and August (the end of gametogenesis; T3A and T4A, respectively) 2010 (Fig. 1). In total, 56 ramets (n = 7 ramets per treatment for each collecting date) from 12 colonies per date (each colony containing ramets of one or more treatments) were collected; however, a total of 112 ramets, belonging to 24 colonies, were marked at the beginning of the study. This approach was used as a precaution to ensure the availability of additional ramets during the study in case of manipulation effects or natural disturbances. The colonies used to sample ramets were at least 2 m distant from each other. For each treatment, the ramets were identified using four different tags that were attached to the corals with plastic cable ties at the base of the ramets, with stainless steel wire used to tie up the tags [32]. None of the tags or wires was in contact with the coral tissue. Control ramets were tagged to verify that FTA and CCA contact with the coral persisted throughout the experimental period. At the time of collection, each experimental ramet was chiseled underwater and fragmented into three parts. Two fragments (portions representing 1/2 and 1/4 of each ramet) were used to evaluate other biological parameters of *O*. *annularis* as reported by [32], and the third fragment (1/4 part of each ramet) was further chiseled underwater to collect coral pieces of ~5 cm^2^ obtained from the central upper part of each ramet, ≥ 7 cm distant from the ramet periphery. These specimens were stored in 10% formalin in seawater and transported to the *Instituto de Investigaciones Oceanológicas* (IIO) of the *Universidad Autónoma de Baja California* (UABC) for histological analysis.

### Sample processing

In the laboratory, coral fragments were cut with a hacksaw into 4 cm^2^ squares and rinsed with filtered seawater. Clean fragments were immersed in Zenker solution for 18 h to strengthen the coral tissue. The fragments were rinsed continuously for 7.5 h with a gentle flow of freshwater to prevent damage to the tissue. The fragments were then immersed in a decalcifying solution of 10% HCl for a period of no less than 5 h, and the solution was replaced several times during this period. Later, the tissue was dehydrated in ethanol at various concentrations (70–100%), immersed in Hemo-De, and finally embedded in paraffin (~ 20 h). Paraffin blocks were placed in a manual rotary microtome to obtain longitudinal sections. For each sample, per treatment and collection date (n = 56), we obtained 10 slides (560 slides in total) containing one ribbon (a group of approximately 6 sections or cuttings of 7 μm in thickness). The distance between each group of cuttings was greater than 250 μm to ensure that the information obtained was from different polyps. The tissue was stained with Mallory-Heidenhain stain and fixed permanently for subsequent observation. The sample processing was similar to that described by [42].

Permanent preparations were observed through stereoscopic and compound Olympus microscopes, and photomicrographs were obtained using an AxioCam in a Carl Zeiss microscope and a Canon PowerShot G9 in an Olympus compound microscope. The stages of the male and female gametes were recorded, and the maximum diameter of the eggs (i.e., egg size) was measured with a micrometer ruler. The mean percentage of developmental stages of gametes and the maximum diameter of all the eggs found were calculated from at least 10 polyps (a polyp per slide) of each sample per treatment at the beginning (~ 280 polyps) and the end (~ 280 polyps) of gametogenesis. In May (when spermaries were absent), the percentage of each female stage was obtained from the total number of oocytes observed in the samples, whereas in August (with spermaries present), the percentage of the stages for both gametes was obtained from the total oocytes and spermaries observed in the samples. For each oocyte, the stage of the contiguous spermary was recorded.

### Description of gametogenic stages of *O*. *annularis*

**Fig 2:**
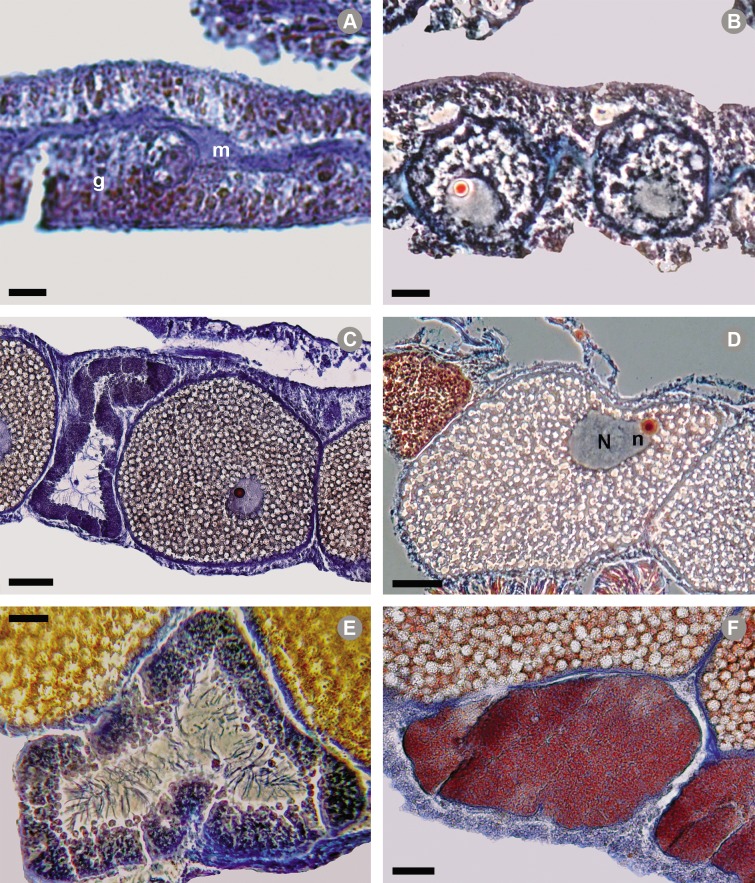
*Orbicella annularis* gametogenesis. A) Stage I oocyte located between the mesoglea (m) and the gastrodermis (g); B) Stage II oocyte; C) Stage IV oocyte (next to a Stage III spermary); D) Stage V oocyte with nucleus (N) and nucleolus (red small circle) in its periphery; E) Stage III spermary with spermatocytes surrounding its periphery; and F) Stage IV spermary with spermatocytes homogeneously distributed. Microphotographs: NP Cetz-Navarro. Scales: A = 10 μm; B = 20 μm; C = 75 μm; D and E = 50 μm; F = 25 μm.

The maturation stages of female and male gametes of *Favia fragum* described by [43] were used to establish the gametogenic stages of *O*. *annularis*. We considered five maturation stages for oocytes and four for spermaries. In oogenesis, Stage I presented small oocytes (17.8 ± 6.8 SD μm in diameter), oval and immersed mainly in the mesoglea, with little cytoplasm and a blue nucleus (Fig. 2A). Stage II had larger oocytes (70.9 ± 19.9 μm in diameter) with evidence of the onset of vitellogenesis. The nucleus and nucleolus were distinguished, and all structures were stained blue except the nucleolus (reddish orange); at this stage, the oocytes were aligned inside the mesenteries (Fig. 2B). Stage III oocytes were those that had the nucleus located in their central portion and that had completed vitellogenesis. Stage IV presented reddish or slightly bluish oocytes (275.4 ± 22.4 μm in diameter), with the nucleus migrating toward the periphery, i.e., between the center and the periphery (Fig. 2C). Stage V showed reddish mature eggs (323.0 ± 28.7 μm in diameter) and the nucleus on the periphery and showing a slight indentation (Fig. 2D). In spermatogenesis, Stages I and II were the same as those described by [43] for *Favia fragum*. Stage III had blue spermaries that contained spermatocytes surrounding the periphery and with a large central cavity; the heads measured ~5 μm in diameter (Fig. 2E). Stage IV presented reddish spermaries with mature spermatozoids that were homogeneously distributed and with heads ~2 μm in diameter (Fig. 2F).

### Statistical analyses

The mean percentage values of gamete stages found in May (oocytes = 100%) and August (oocytes + spermaries = 100%) were subjected to a one-way permutational multivariate analysis of variance (PERMANOVA; factor: treatment) using Type III (partial) sums of squares and unrestricted permutation of raw data on a Euclidean distance matrix with 999 permutations to assess differences among treatments. A similarity percentage (SIMPER) analysis was used to observe the contribution made to the dissimilarity among treatments by the percentage of gamete stages. Both analyses were performed with the statistical software PRIMER 6 and PERMANOVA+ [44]. The mean values of the maximum diameter of the eggs at the beginning and the end of gametogenesis were analyzed with a nonparametric Kruskal-Wallis test (factor: treatment) because the data were not normally distributed (Shapiro-Wilk test); later, a Dunn’s test was applied to compare the mean values for pairs of treatments.

## Results

### Percentage of gametogenic stages of *O*. *annularis* at the beginning and the end of the experimental period

**Fig 3:**
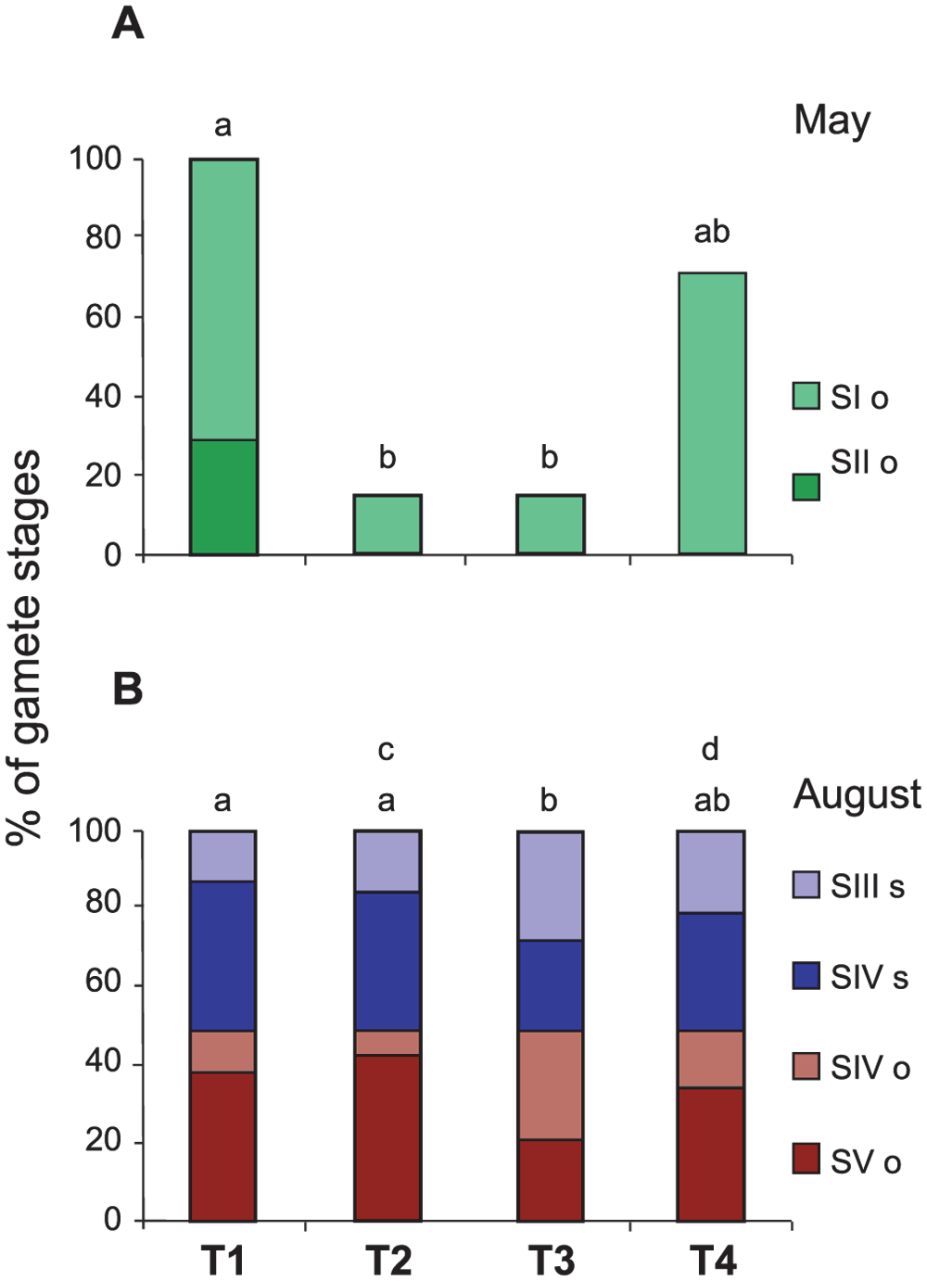
Mean percentage values of gamete stages of *Orbicella annularis* during gametogenesis. A) Gamete stages found in May, the beginning of gametogenesis: Stage I oocytes (SI o) + Stage II oocytes (SII o) = 100%, and B) gamete stages found in August, the end of gametogenesis: Stage IV oocytes (SIV o) + Stage V oocytes (SV o) + Stage III spermaries (SIII s) + Stage IV spermaries (SIV s) = 100%. Treatments: T1) ramets with long-term removal of FTA, T2) ramets with short-term removal of FTA, T3) control ramets always surrounded by FTA, and T4) control ramets always surrounded by CCA. Lower-case letters (a-b for May, and a-b and c-d for August) above pair of bars indicate treatments that were significantly different (P ≤ 0.005). n = 7 collected ramets per treatment on each date.

The development of gametes of *O*. *annularis* showed differences among treatments at the beginning (PERMANOVA, Pseudo-F = 4.36, P-*perm* = 0.005) and at the end (Pseudo-F = 4.62, P-*perm* = 0.004) of the experimental period (Fig. 3). At the beginning of gametogenesis (May), only female gametes of Stages I and II were found. T1M (with long-term removal of FTA) showed a high percentage of Stage I oocytes, and Stage II oocytes were observed only in this treatment. In contrast, T2M (with short-term removal of FTA) and T3M (with FTA bordering coral tissue throughout the experimental period) showed a lower percentage of Stage I oocytes. T4M (with coral tissue surrounded by CCA) yielded results similar to those of other treatments. Specifically, the percentages of Stage I oocytes were similar to those in T1M, whereas no Stage II oocytes were observed (this absence of Stage II oocytes was consistent with the findings for T2M and T3M). The ramets in treatments with short-term algal removal (T2M) had oocyte development similar to the ramets that were surrounded by FTA throughout the experimental period (T3M) (Fig. 3A). At the end of gametogenesis (August), both female and male gametes were present: Stages IV and V of female gametes and Stages III and IV of male gametes. Stage III of oocytes and Stages I and II of spermaries were not observed in August, most likely because these stages developed between the two collection dates. Ramets of T1A and T2A (with long- and short-term removal of FTA, respectively) had a higher percentage of mature than of immature gametes, contrasting with the results for gametes of ramets of T3A bordered by FTA; i.e., the ramets of T1A and T2A had a higher percentage of Stage V oocytes and Stage IV spermaries (both mature) and a lower percentage of Stage IV oocytes and Stage III spermaries (both immature) than the gametes of ramets of T3A. An additional difference was found between T2A and T4A, namely, T4A showed higher percentages of Stage IV oocytes and Stage III spermaries, both immature, than T2A (Fig. 3B).

### Maximum diameter of eggs (oocytes) of *O*. *annularis* at the beginning and the end of gametogenesis

**Fig 4:**
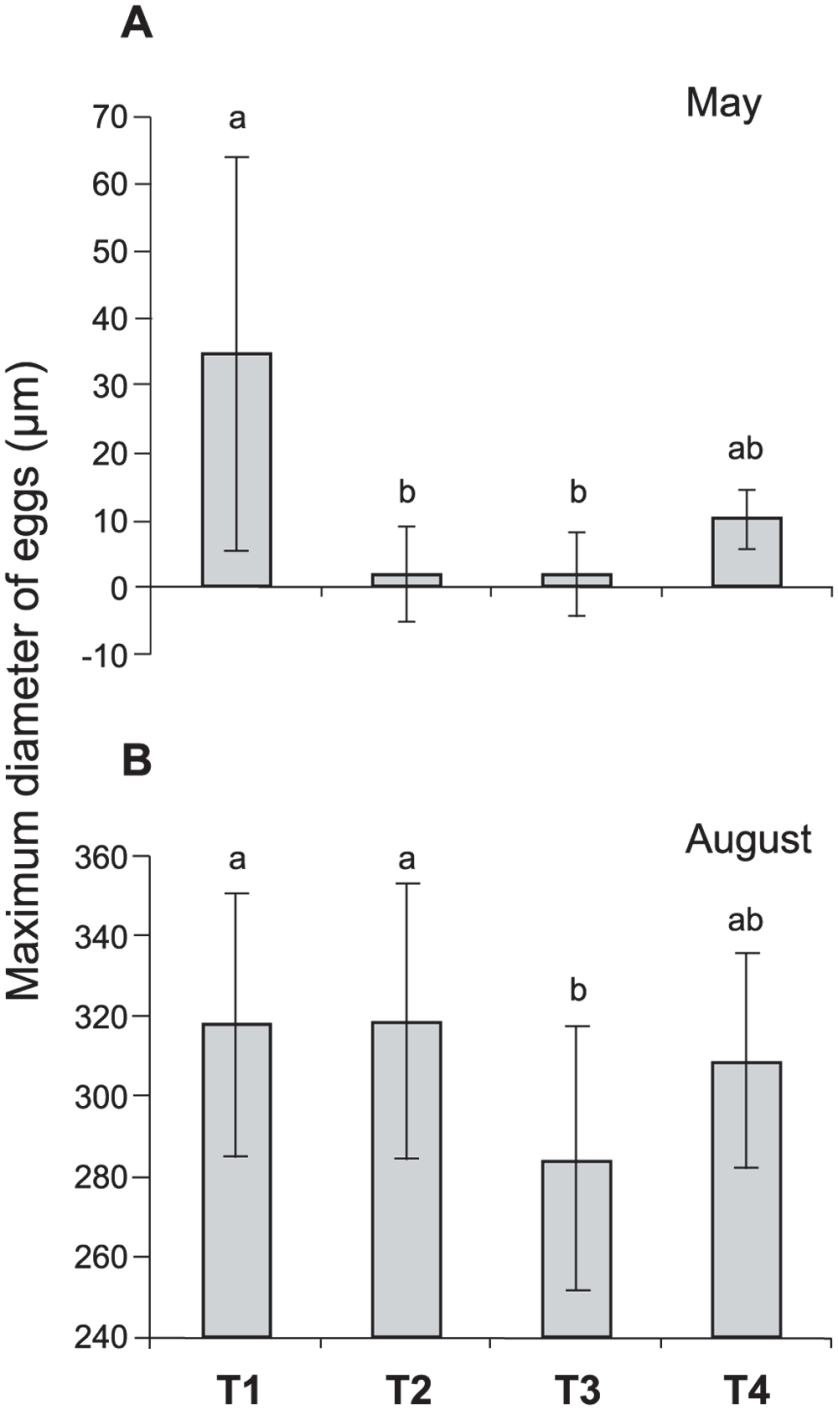
Mean values ± SD of the maximum diameter of eggs of *Orbicella annularis* during gametogenesis. A) Egg size at the beginning (May) of gametogenesis, and B) egg size at the end (August) of gametogenesis. Treatments: T1) ramets with long-term removal of FTA, T2) ramets with short-term removal of FTA, T3) control ramets always surrounded by FTA, and T4) control ramets always surrounded by CCA. Lower-case letters (a-b) above pair of bars indicate treatments that were significantly different (P = 0.000). n = 7 collected ramets per treatment on each date.

The maximum diameter of eggs (MDE) or egg size of *O*. *annularis* showed differences among treatments at the beginning (Kruskal-Wallis test, χ^2^ = 20.97, df = 3, P = 0.000) and at the end (χ^2^ = 18.92, df = 3, P = 0.000) of gametogenesis (Fig. 4). At the beginning of gametogenesis (May), the MDE of T1 was larger than that of the oocytes of T2 and T3 (Dunn’s test, P < 0.05), whereas the oocytes of T4 were similar in diameter to those in the other treatments (Fig. 4A). At the end of gametogenesis (August), the MDE of T1 and T2 was larger than that of oocytes at T3 (Dunn’s test, P < 0.05), whereas the MDE of T4 was similar to that of the other treatments (Fig. 4B).

## Discussion

The removal of FTA from the periphery of *Orbicella annularis* tissue allowed a better development of gametes in terms of the MDE and PGS of oocytes, most likely because the stress was reduced when the FTA were removed [32,33], allowing the coral to re-allocate more resources to reproduction. Ramets subjected to long- (T1 in May and August) and short (T2 in August)-term removal of algae had larger eggs and a higher percentage of mature gametes (PMG) than ramets that were surrounded by FTA throughout the experimental period (T3). In contrast, ramets with FTA, having smaller eggs and lower PMG, most likely diverted resources from reproduction to maintenance, including the defense of polyps that were interacting with the algae [33] in the peripheral part of the ramets. A decrease in egg size, as produced in the presence of FTA, also occurs when other stressors affect corals. Such stressors include bleaching [45], increased seawater temperatures due to El Niño events [42], nutrient enrichment of seawater [46,47], and sewage pollution [48]. If *O*. *annularis* egg size is more sensitive (than the number of eggs per mesentery and the number of mesenteries with eggs per polyp) to sub-lethal stress caused by algal presence [33], then the ramets of T1 represent the lowest stress condition caused by FTA removal. A similar reduction in stress condition was also registered in terms of PGS, with a higher development of gametes in the ramets of T1. Nevertheless, the PGS is proposed here as a better estimator of sub-lethal algal stress than egg size because the PGS revealed differences between treatments (T2 *vs*. T4 in August) not detected by the analysis of egg size.

The response of gametes in ramets bordered by CCA (T4), with oocyte size and PGS similar to the ramets of T3, was unexpected because the same ramets were less stressed when the stress was evaluated through tissue thickness, chlorophyll *a* per square centimeter, zooxanthellae density, and mitotic index [32]. CCA presence in coral reefs is generally considered beneficial [17–20,32]; nevertheless, some CCA can overgrow recruits, eliminate recruits by sloughing of outer cell layers [17,49,50], and overgrow adult corals [51,52]. Because we marked the reproductively mature ramets with a plastic cable tie at their base for use as a reference point for measuring the distance to the periphery of the coral tissue, we consider that the overgrowth of coral tissue was null or negligible, most likely because the ramets were sufficiently large to avoid overgrowth by CCA [53]. However, the responses of the ramets surrounded by CCA, similar to the ramets with FTA (but also similar to the ramets with FTA removal), suggest that the crustose algae had unknown negative effects on the reproductive performance of *O*. *annularis*. Accordingly, our results indicate that assessing sub-lethal algal stress through coral reproduction metrics (e.g., [33], this study) is a more sensitive method than the use of other [32] biological parameters; also, to focus on the negative effects of stress on reproduction can be more relevant to the research questions because these effects impact the fitness of *O*. *annularis*.

The effect of FTA removal on the development of gametes, both in terms of MDE and in terms of PGS, differed between short- and long-term removal in May but not in August. In May, only one of seven ramets surrounded by FTA (T3) presented eggs (Stage I oocytes), but all ramets with long-term removal of FTA (T1) had eggs. These results were similar to the findings of [54], who, in May, found no oocytes in *O*. *annularis* colonies stressed by bleaching, unlike unstressed colonies, which exhibited oocytes. The beneficial effect of FTA removal (T1 and T2) on reproduction occurs before the spawning of gametes in August, at 2.5 months or, most likely, before that time, as previously observed in relationship to the reduction of stress on *O*. *annularis* in terms of fecundity [33] and other biological parameters [32]. The relatively rapid recovery of gamete development after the removal of the stressing FTA in August contrasts with the long-lasting or severe negative effects on reproduction caused by the stress of coral bleaching and other stressors. For example, corals affected by bleaching events can show a lack of sexual reproduction after 1.5 years [55], although the susceptibility to bleaching is species-specific, as observed in the percentage of mortality of bleached gravid colonies of two *Acropora* species [24]. Similarly, coral tissue infection by diseases was found to prevent the formation of eggs in *O*. *faveolata* [30], and it has been suggested that an El Niño event prevented the formation of mature stages of gametes in *Pocillopora damicornis* for over a year [42]. In the case of *O*. *annularis*, [54] reported that bleaching stopped gametogenesis, and subsequent studies showed that some colonies continued to show reproductive failure even one year after a bleaching event; after two years, small mesenteries with eggs were still produced [56].

The relatively rapid relaxation of negative effects on gamete development after the removal of FTA in August suggests that the negative effects of these algal assemblages on coral reproduction have only a short-term impact. Our results agree with those [33] reporting the beneficial effect of short-term removal of turf+macroalgae on the fecundity of *O*. *annularis*. However, turf algae and macroalgae are most likely involved in long-term, chronic, deleterious impacts on coral reproductive responses due to their increased abundance and ubiquity during recent decades in the Caribbean [8,57–60], which would promote the frequency and duration of coral-algal interactions [12]. In these interactions, algae can play significant roles and affect corals through several mechanisms, e.g., overgrowth, shading, allelopathy, smothering and burial of coral tissue [31,32,61]. Notably, algal cover has increased at the same pace at which the population of *O*. *annularis* has declined in different areas of the Caribbean in recent decades [62–68]. The stress on coral reproductive parameters caused by the presence of FTA and macroalgae ([33–36], this study) could be included in the long-term stressors that are compromising the presence of *O*. *annularis* in this region.

If spawning of *O*. *annularis* occurs 6 to 8 days after the full moons of August-October [34,40], and if we consider that we collected ramets the day of the full moon of August 24, a few days before a potential spawning event, the ramets that had gametes with the highest probability of fertilization success were those with FTA removal (T1 and T2). The percentage of mature gametes in these treatments was, on average, almost double (83% of Stage V oocytes and 72% of Stage IV spermaries) that of those ramets surrounded by FTA (44% of Stage V oocytes and 45% of Stage IV spermaries). Low PMG in the ramets of *O*. *annularis* surrounded by FTA may indicate that they could produce two spawning events (in August and September) by extending their oogenesis and spermatogenesis. Nothing is known about detrimental effects of algae during temporal extensions of coral gametogenesis. However, other stressors affect reproductive effort in corals. For example, when *Porites porites* colonies were stressed by eutrophication, their reproductive season was 1 to 2 months longer than at relatively unpolluted sites; however, their reproductive effort (in terms of number of larvae per area of coral tissue) was lower than in colonies from unpolluted sites [22]. Alternatively, *O*. *annularis* ramets surrounded by FTA could show only one spawning event, in August, with the remnant gametes being resorbed.

In summary, the negative effects on gamete development due to the stress produced by FTA in *O*. *annularis* is similar to, although apparently less severe than, other stressors affecting corals. Thus, algae, primarily under the deteriorating conditions of Caribbean coral reefs, are added to the series of stressors (bleaching, increases in water temperature, diseases, suspended sediment in water, pollution and eutrophication) that can affect the sexual reproduction of hermatypic corals. Low recruitment of the *O*. *annularis* species complex has been reported throughout the Caribbean [37,69,70] and has been virtually absent at certain sites over several years of observation [64,65,71]. For this reason, it has been suggested that *O*. *annularis* populations will continue declining and will not recover because recruitment is low [66] or that their recovery will take a century or more [64]. Considering that *O*. *annularis* populations are declining, that several stressors, including algae, are stressing the remaining colonies and limiting their sexual reproduction, and that their recruitment is low or nonexistent, the future scenario for the reef builder *O*. *annularis* species complex in the Caribbean appears uncertain.
